# Factors associated with difficulty accessing health care for infants in Canada: mothers’ reports from the cross-sectional Maternity Experiences Survey

**DOI:** 10.1186/s12887-016-0733-4

**Published:** 2016-11-25

**Authors:** Alisa D. Brandon, Christy Costanian, Manal F. El Sayed, Hala Tamim

**Affiliations:** 1 School of Kinesiology and Health Science, York University, Toronto, ON Canada; 2 Division of Neonatology, Department of Pediatrics, McMaster University, Hamilton, ON Canada

**Keywords:** Health care access, Canada, Infants, Prevalence, Predictors

## Abstract

**Background:**

Approximately 13% of Canadian mothers report difficulty accessing health care for their infants, yet little is known about the factors associated with difficulty. Therefore, we examined factors associated with difficulty accessing non-routine health care for Canadian infants, from birth to 14 months of age, as reported by their mothers.

**Methods:**

Data was drawn from the Maternity Experiences Survey (MES), a cross-sectional, nationally representative survey of mothers who gave birth between November 2005 and May 2006, aged 15 years or older, and lived with their infants at the time of survey administration. A multivariable logistic regression analysis was conducted to determine factors associated with reporting difficulty, with difficulty defined as a mother reporting it being somewhat or very difficult to access a health care provider.

**Results:**

Analysis of 2832 mothers who reported needing to access a health care provider for their infant for a non-routine visit found that 13% reported difficulty accessing a provider. Factors associated with reporting difficulty were: residing in Quebec (aOR 1.89, 95% CI: 1.31–2.73), being an immigrant (aOR 1.58, 95% CI: 1.10–2.27), mistimed pregnancy (aOR 1.44, 95% CI: 1.05–1.98), low level of social support (aOR 1.69, 95% CI: 1.05–2.73), good health (aOR 1.88, 95% CI: 1.43–2.47), postpartum depression symptoms (aOR 1.55, 95% CI: 1.02–2.37) and a self-reported ‘too-short’ postpartum hospital stay (aOR 1.69, 95% CI: 1.21–2.35). Additionally, accessing care for an infant with a birth weight of 2500 g or more (aOR 2.43, 95% CI: 1.02–5.82), was associated with reporting difficulty. Household income, mothers’ level of education, marital status, Aboriginal ethnicity, and size of community of residence were not associated with difficulty accessing care.

**Conclusions:**

Ease of health care access for Canadian infants is not equal, suggesting that efforts to improve access should be tailored to groups facing increased difficulties.

## Background

Easy and universal access to health care services is essential to children’s health [1]. Furthermore, unhindered access to health services is of notable importance for infants, as the mortality rate for infants (children under one year of age) is the highest among all childhood age groups in Canada [2]. Moreover, while Canadian infant mortality rate demonstrated a sharp decline between the mid 1960s and 1990s [3], substantial decreases have not been seen since the mid 1990s, resulting in Canada’s fall from 5th to 28th place in infant mortality rankings among the 35 OECD (Organization for Economic Co-operation and Development) countries as of 2012 [4]. However, there is some evidence that improved access to health care may lead to decreased infant mortality. For example, after adjustment, one study found that American infants who did not participate in either Medicaid or private insurance were 1.39 (95% CI: 1.04–1.86) times more likely to die from perinatal conditions and 1.46 (95% CI: 0.97–2.20) times more likely to die from non-perinatal conditions, injuries, and infections [5]. Similarly, primary care physician density has been shown to have an independent inverse association with infant mortality in Canada, the United States, and the European Union [6–8], suggesting that easier access to primary care may result in lower infant mortality.

The Canadian Health Care Act requires provinces to provide health services to all residents other than small subpopulations such as members of the armed forces, individuals residing in penitentiaries, and individuals who have not completed a minimum residence period in Canada. Provinces must also ensure that access to health services is unhindered by barriers such as age, health status or income [9]. Under this act, parents or other caregivers should be able to access, without hindrance, health services on behalf of virtually all infants born in Canada. Despite the stipulations of the Health Care Act, a previous report based on the 2006–2007 Maternity Experiences Survey (MES) revealed that 13% of mothers reported difficulty accessing health care for their infants. The prevalence of reporting difficulty varied by province, from only 6.8% of Saskatchewan mothers to 18.8% of Northwest Territories mothers reporting difficulty [9].

To our knowledge, only three studies have examined predictors of Canadian children’s poor health care access. Two of these studies, based on Toronto [10] and Alberta children [11], propose that inferior access to health care may be associated with low socioeconomic status, while a third study, based on Ontario children [12], documented an association between poor health care access and low primary care physician density. However, studies from other nations with single payer systems illuminate other characteristics that may be associated with difficulty accessing health care services for children. Studies based in the United Kingdom [13] and Nordic Europe (Finland, Denmark, Sweden, Iceland, & Norway) [14] suggest that socioeconomic status is not associated with children’s health care access except for the most disadvantaged groups (parents with only primary education), while a further study based on the Maternity Experiences Survey did not find an independent relationship between socioeconomic status and inadequate prenatal care among Canadian mothers [15]. However, maternal characteristics such as being an immigrant, young maternal age, single relationship status, and Aboriginal ethnicity have been associated with poor health care access in countries with single-payer health systems [16–18]. The previously mentioned study based on the Maternity Experiences Survey also found that immigrant mothers were more likely to report inadequate prenatal care [15].

Despite the potential benefits of improving Canadian infants’ health care access and the high percentage of Canadian mothers who report struggling to access health care for their infants, no national Canadian study has examined the factors associated with mothers reporting difficulty accessing health care for their infants. Therefore, the objective of this study was to examine the characteristics associated with mothers’ reporting difficulty in accessing non-routine health care for their infants.

## Methods

### Study design and data collection

This study used data from the Maternity Experiences Survey (MES). The design and methods of the MES have been described in further detail elsewhere [19]. The MES included a nationally representative, random sample of mothers who delivered single live infants while aged 15 years or older between February 15, 2006 and May 15, 2006 in the Canadian provinces or November 1, 2005 to February 1, 2006 in the Canadian Territories and lived with their infant at the time of survey administration. Mothers living on First Nations (American Indian) reserves or collective dwellings (hospital, nursing home, rooming house, military base, group home etc.) at the time of survey administration were not eligible to participate in the MES. Mothers were sampled from the unedited 2006 Canadian census. Of the 8244 mothers selected to complete the survey and deemed eligible, 6421 mothers completed the survey, resulting in a participation rate of 78%. Interviews were conducted in 15 languages, including English and French, using a Computer Assisted Telephone Interview (CATI) technique. Interviews were conducted when infants were aged 5 to 14 months [19]. The MES research protocol was reviewed by the Health Canada’s Science Advisory Board and Research Ethics Board and the Federal Privacy Commissioner, and approved by the Statistics Canada’s Policy Committee. Ethics approval for this analysis was not needed as this was based on a secondary analysis of the MES collected by Statistics Canada. Access to the MES database was obtained through the Research Data Centre in Toronto, Canada.

### Measures

*For the current project*, data analysis was restricted to mothers who reported that their infant needed to see a health care provider for a non-routine problem or illness. This information was collected by the MES asking mothers “Since he/she was born, has ^baby’s name needed to see a doctor or other healthcare provider for a problem or illness other than a routine check-up?”. The main outcome of this study was mother’s reported level of difficulty when attempting to see a health care provider for her infant, for a non-routine problem or an illness, from birth until a maximum of 14 months of age. Specifically, in the MES, mothers were asked: “Overall, how easy or difficult was it to see a healthcare provider for ^baby’s name?” This outcome was dichotomized into: “difficult” (includes responses of “very difficult” or “somewhat difficult”) and “not difficult” (includes responses of “very easy”, “somewhat easy” or “neither easy nor difficult”). Independent variables included socioeconomic, demographic, maternal, health service and infant factors. Socioeconomic factors included total household income and education. Demographic factors included region of residence, immigration status, self-identified Aboriginal ethnicity, marital status, maternal age, and size of community of residence. Maternal factors included timing of pregnancy, total number of live births, level of postpartum social support, mother’s perceived health, and postpartum depression symptoms as measured by the Edinburgh Postpartum Depression Scale (EPDS). Health service factors included mother receiving prenatal care in a language she could understand, feelings about length of postpartum hospital stay, and type of prenatal care provider. The variable prenatal care provider was dichotomized into “family physician” and “other”, to determine if receiving prenatal care from a family physician would result in easier access to primary care for the infant. The infant factor was infant’s weight at birth. The outcome and the independent variables (factors) were collected through self-report.

### Statistical analysis

**Fig. 1:**
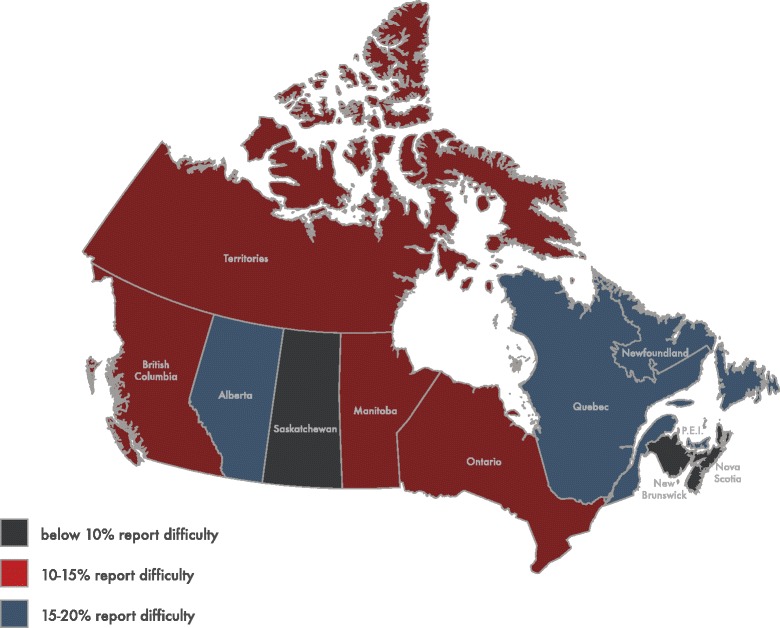
Prevalence rates of mothers reporting difficulty accessing a health care provider for their infant across the Canadian provinces and territories* (2005/06). * All three Canadian territories (Yukon, The North West Territories and Nunavut) merged to prevent unweighted cell counts below five. Depicts the prevalence rates of reporting difficulty accessing non-routine health care for an infant by Canadian province and territory grouped as: below 10% report difficulty, 10 to 15% report difficulty, and 15 to 20% report difficulty

First, descriptive analyses of both the outcome and the independent variables (factors) were conducted. Next, bivariate associations between each factor and reporting difficulty accessing health care for an infant were determined through chi squared tests. Bivariate associations between region of residence and reporting difficulty were determined on both a provincial level (see Fig. 1) and a regional level (included in logistic regression model). Next, to ascertain which factors were independently associated with reporting difficulty, we created a multivariable logistic regression model with the outcome being reporting difficulty accessing health care for an infant, and all factors added as independent variables. We applied population weights to all estimates to make our results representative of the target population at the time of the survey. To account for the complex sampling design, bootstrapping was performed to calculate the 95% CI estimates. Population weights and bootstrap weights were all created by Statistics Canada and provided with the MES data file. All analyses were conducted with Stata Data Analysis and Statistical Software (Stata, version 13.0).

## Results

After excluding mothers whose infants did not need to see a health care provider for a problem or illness (49% of all mothers), a sample size of 2832 remained. Missing data on the main outcome variable did not exceed 5%. Around 63% of women found it very easy to obtain access to a health care provider for their infant, whereas slightly less than one fifth of women found it somewhat easy. Almost 4% of women reported that it was neither easy nor difficult, and around 8% found it somewhat difficult, while 5% stated that it was very difficult to see a health care provider for their ill infant (results not shown). After combining the latter two categories into one category labeled as “difficult”, 13% reported difficulty accessing a health care provider for their infant (results not shown). Figure 1 demonstrates the prevalence of reporting difficulty accessing a health care provider for an infant by province and territory. Newfoundland and Labrador, Quebec and Alberta demonstrated the highest prevalence of mothers reporting difficulty, with each province or territory having a prevalence of 15% or higher. On the other hand, New Brunswick and Saskatchewan had the lowest prevalence rates, at less than 10%.

Table 1 presents the distribution of characteristics (factors) of mothers who reported difficulty accessing health care for their infants, and the unadjusted and adjusted odds ratios (ORs) of these factors. While nine factors were associated with reporting difficulty at the bivariate level, mothers with certain characteristics were especially likely to report difficulty. For example, 25.3% of mothers who reported social support none or little of the time also reported difficulty accessing health care. Furthermore, mothers who reported good health (prevalence rate of difficulty: 19.8%), fair/poor health (18.5%), and had postpartum depression symptoms (21.7%) also had especially high prevalence rates of reporting difficulty. Of the nine associations that were statistically significant during bivariate analysis, all except maternal age at birth and fair/poor health remained statistically significant after adjustment. After adjustment, mothers who resided in Quebec (aOR = 1.89, 95% CI: 1.31–2.73), were immigrants (aOR = 1.58, 95% CI: 1.10–2.27), had a mistimed pregnancy (aOR = 1.44, 95% CI: 1.05–1.98), had postpartum social support none or little of the time (aOR = 1.69, 95% CI: 1.05–2.73), reported their health as good (aOR = 1.88, 95% CI: 1.43–2.47), had an Edinburgh Postnatal Depression Score of 13 or higher (aOR = 1.55, 95% CI: 1.02–2.37), and felt that their postpartum hospital stay was ‘too short’ (aOR = 1.69, 95% CI: 1.21–2.35) were at an increased odds of reporting difficulty accessing health care for their infant. Lastly, while infant’s birth weight was not significant in the unadjusted analysis, a birth weight of 2500 g or heavier became significantly associated with reporting difficulty in the adjusted model (aOR = 2.43, 95% CI: 1.02–5.82).

**Table 1:** Unadjusted and adjusted associations between various factors and reporting difficulty accessing health care for an infant (*n* = 2832)

	Reported difficulty	Reported no difficulty	Unadjusted OR^a^ (95% CI)^b^	Adjusted OR^a^ (95% CI)^b^
	N (%)	N (%)		
Socioeconomic factors				
Household income (CAD)				
≥ $80,000	128 (11.9)	945 (88.1)	1.00	1.00
$40,000–$79,999	158 (12.9)	1064 (87.1)	1.10 (0.84–1.43)	0.89 (0.66–1.21)
< $40,000	111 (14.7)	645 (85.3)	1.28 (0.98–1.67)	0.98 (0.67–1.43)
Mother’s highest level of education				
Degree or higher	151 (13.1)	999 (86.9)	1.00	1.00
Some Postsecondary	181 (12.4)	1273 (87.6)	1.07 (0.81–1.43)	0.89 (0.65–1.20)
High school or less	86 (14.0)	527 (86.0)	0.94 (0.73–1.21)	0.96 (0.64–1.43)
Demographic factors				
Region of residence				
Atlantic	24 (10.4)	208 (89.6)	1.00	1.00
Quebec	140 (18.3)	624 (81.7)	**1.92 (1.42–2.60)**	**1.89 (1.31–2.73)**
Ontario	121 (10.2)	1065 (89.8)	0.98 (0.72–1.32)	0.92 (0.64–1.33)
Prairies	84 (13.3)	547 (86.7)	1.32 (0.95–1.82)	1.43 (0.97–2.11)
British Columbia	51 (12.4)	356 (87.6)	1.22 (0.82–1.82)	1.16 (0.72–1.88)
Territories	3 (14.0)	16 (86.0)	1.39 (0.94–2.06)	1.13 (0.66–1.94)
Immigrant				
No	320 (12.1)	2336 (87.9)	1.00	1.00
Yes	100 (17.5)	471 (82.5)	**1.54 (1.17–2.03)**	**1.58 (1.10–2.27)**
Aboriginal				
No	404 (13.1)	2678 (86.9)	1.00	1.00
Yes	19 (13.3)	124 (86.7)	1.02 (0.64–1.62)	1.04 (0.58–1.85)
Marital status				
Has a partner	386 (13.1)	2552 (86.9)	1.00	1.00
No partner	36 (12.2)	256 (87.8)	0.92 (0.64–1.32)	0.85 (0.51–1.41)
Maternal age				
≥ 35	56 (10.5)	479 (89.5)	1.00	1.00
20–34	346 (13.4)	2231 (86.6)	1.32 (0.96–1.83)	1.30 (0.90–1.87)
< 20	19 (18.0)	88 (82.0)	**1.87 (1.11–3.15)**	1.65 (0.76–3.60)
Size of community of residence				
Rural population	68 (12.0)	500 (88)	1.00	1.00
< 500,000	152 (12.5)	1066 (87.5)	1.04 (0.76–1.42)	1.22 (0.85–1.75)
≥ 500,000	192 (14.2)	1163 (85.8)	1.21 (0.89–1.64)	0.97 (0.66–1.43)
Maternal factors				
Timing of pregnancy				
Well-timed	287 (12.0)	2100 (88.0)	1.00	1.00
Mistimed	110 (17.3)	527 (82.7)	**1.52 (1.17–1.98)**	**1.44 (1.05–1.98)**
Unwanted	22 (11.2)	171 (88.8)	0.92 (0.56–1.51)	0.85 (0.47–1.56)
Total number of live births				
More than one	216 (12.6)	1498 (87.4)	1.00	1.00
One	207 (13.6)	310 (86.4)	1.09 (0.88–1.35)	1.02 (0.79–1.32)
Postpartum social support				
Most of the time	327 (11.9)	2412 (88.1)	1.00	1.00
Some of the time	52 (16.0)	275 (84.0)	1.41 (1.00–1.99)	1.24 (0.82–1.88)
None/little of the time	43 (25.3)	127 (74.7)	**2.50 (1.68–3.72)**	**1.69 (1.05–2.73)**
Mother’s perceived health				
Excellent/very good	244 (10.5)	2076 (89.5)	1.00	1.00
Good	146 (19.8)	592 (80.2)	**2.10 (1.65–2.66)**	**1.88 (1.43–2.47)**
Fair/poor	34 (18.5)	148 (81.5)	**1.93 (1.27–2.93)**	1.60 (0.98–2.60)
Edinburgh Postnatal Depression Score				
0–12	365 (12.3)	2601 (87.7)	1.00	1.00
≥ 13	52 (21.7)	188 (78.3)	**1.98 (1.40–2.79)**	**1.55 (1.02–2.37)**
Health service factors				
Prenatal care given in a language mother could understand				
Yes	407 (12.9)	2752 (87.1)	1.00	1.00
No	16 (23.0)	53 (77.0)	2.01 (0.96–4.20)	1.16 (0.43–3.15)
Feelings about length of hospital stay				
About right	264 (12.1)	1925 (87.9)	1.00	1.00
Too short	82 (17.7)	382 (82.3)	**1.57 (1.17–2.12)**	**1.69 (1.21–2.35)**
Too long	73 (13.1)	482 (86.9)	1.10 (0.80–1.50)	0.96 (0.67–1.37)
Prenatal care provider				
Family physician	135 (11.7)	1019 (88.3)	1.00	1.00
Other	287 (14.0)	1768 (86.0)	1.23 (0.97–1.55)	1.27 (0.96–1.67)
Infant related factors				
Birth weight (grams)				
< 2500	12 (7.80)	147 (92.2)	1.00	1.00
≥ 2500	410 (13.4)	2661 (86.6)	1.82 (0.90–3.66)	**2.43 (1.02–5.82)**

^a^Sample size estimated using normalized weights

^b^95%Confidence Interval was calculated using bootstrapping technique

Bolded ratios are significant

## Discussion

This was the first national, population-based study to examine factors associated with mothers reporting difficulty accessing health care for their infants. Our study revealed that 13% of Canadian mothers reported difficulty accessing health care for their infants. Furthermore, the results document that in Canada’s universal health system, mothers who report difficulty accessing health care for their infants are markedly different from mothers who do not report difficulty. After adjustment, maternal factors significantly associated with report of difficulty were: living in Quebec, being an immigrant, having a mistimed pregnancy, social support none or little of the time, good health (in comparison to excellent/very good), postpartum depression symptoms, and feeling that the postpartum hospital stay was “too short”. In addition, attempting to access health care for an infant who was 2500 g or heavier at birth was associated with report of difficulty.

Contrary to previous studies that examined children’s health care access in Toronto [10] and Alberta [11], the present study found no association between low socioeconomic status (measured by household income and mother’s highest level of education) and reporting difficulty accessing health care for an infant. However, out results are similar to a recent study based on the MES that reported no association between income, education, and inadequate prenatal care [15]. In addition, a study from the United Kingdom did not find an association between socioeconomic status and children’s (aged 0 to 19 years) health care access [13], while a study based in Northern Europe found that parents’ with primary levels of education had more difficulty accessing health care for their children (aged 2 to 17 years), but socioeconomic status was not associated with health care access past this very disadvantaged group [14]. Our study may not have found an association between socioeconomic status and health care access because the socioeconomic status categories were too broad and combined mothers in very low and low socioeconomic status groups. Furthermore, mothers living in extreme poverty may not have participated in the MES because they may have lacked a telephone and could not be contacted, did not have time to complete the survey due to work obligations, or been distrustful of government agencies.

Compared to mothers residing in the Atlantic provinces, mothers residing in Quebec had about twice the odds of reporting difficulty accessing health care for their infant. This is consistent with reports that show Quebec to be the Canadian province with the highest percentage (24.9%) of residents without a family doctor [18]. Similar to previous research [17], we found that, compared to Canadian born mothers, immigrant mothers had increased odds of reporting difficulty accessing health care for their infant. Possible reasons behind immigrant mothers’ increased rates of reporting difficulty may be fewer relationships in the community, a poor knowledge of the Canadian health care system, and a lack of culturally appropriate care in the community [19], suggesting that a comprehensive strategy, which addresses multiple sources of immigrant mothers’ difficulty, may have the greatest effect on increasing health care access for immigrant women and their children.

Surprisingly, Aboriginal status, single marital status, and younger maternal age were not significantly associated with reporting difficulty accessing health care. In comparison, a Canadian study that compared Aboriginal communities (including on-reserve and off-reserve) versus geographically and socioeconomically matched non-Aboriginal communities in Northern Ontario found that people in Aboriginal communities were significantly more likely to be hospitalized for ambulatory care sensitive conditions, which are indicative of poor health care access [20]. One reason for the discrepancy with the present study is that our use of a subjective measure of health care access may have resulted in mothers comparing the experience of accessing care for their infant with their own experiences accessing health care, which may have been marred by extreme difficulty for Aboriginal mothers who previously lived in Northern Canada or on-reserve. Furthermore, it is important to note that the MES excluded on-reserve Aboriginal mothers, who are more likely to live in extremely remote regions. While young maternal age (<20 years) was not significantly associated with difficulty accessing health care after adjustment, the direction of the association does suggest that teenage mothers may experience increased difficulty in accessing care. The lack of significance may be due to the small number (*N* = 107) of teenage mothers in the present study. In addition to young maternal age and Aboriginal ethnicity, single marital status did not show a significant association with difficulty accessing health care. One possible explanation is that Canadian single mothers are less likely to be employed than married or cohabiting mothers; thus, their schedules may be more flexible in terms of fitting in an appointment with a physician or nurse practitioner [21].

The current study suggests that having a mistimed pregnancy, but not an unwanted pregnancy, is an independent predictor of difficulty accessing health care for an infant. To our knowledge, this is the first study to document such an association. One explanation behind the difference in difficulty accessing care hinges on the vastly different characteristics between women who tend to report unwanted versus mistimed pregnancies. For example, a study based on a large sample of women from 15 American states found that mothers who reported their pregnancies as unwanted, versus mistimed, were more likely to be older, married, higher socioeconomic status, and more likely to have older children [22].

Our study revealed a significant association between a mother having an Edinburgh Postnatal Depression Score of 13 or higher and report of difficulty accessing a health provider for an infant. The Edinburgh Postnatal Depression Scale is the most widely used tool to screen women for perinatal mood disorders, and a score of 13 or higher is considered the optimal cut-off point to screen postpartum women for major depression [23]. The association between postpartum depression symptoms and difficulty accessing health care for an infant is consistent with research from the United States [24], and this difficulty may contribute to the negative effects of postpartum depression on infants’ health and development [25]. Furthermore, this study documented a significant association between experiencing social support little or none of the time and reporting difficulty. This association is consistent with findings from qualitative studies [26–28] that report that low levels of social support may act as a barrier to accessing health care for a child. We also found that mothers’ report of good, as opposed to excellent or very good health, was associated with reporting difficulty accessing health care for an infant. The association between mothers’ report of poor health and difficulty accessing health care did not reach significance, possibly due to the small number of mothers who reported poor health, but the strength of the association does suggest that mothers with poor health suffer from increased difficulty accessing health care for their infants. Health problems may act as a barrier to finding a health care provider, transporting an infant to a health care clinic, and waiting to see a health care provider.

The association between a mother reporting a ‘too short’ postpartum hospital stay and reporting difficulty accessing health care for an infant is consistent with studies that have shown associations between short postpartum hospital stays and neonates’ increased use of emergency rooms [29] and re-admission rates [30]. Mothers who have difficulty accessing primary care may turn to emergency rooms or may forego health care, resulting in progression of illness in the infant and admission to hospital. This consistency lends support to the recommendation, from the Society of Obstetricians and Gynaecologists of Canada, for after birth follow up programs to take into account length of postpartum hospital stay [31]. In addition, follow-up programs may be most effective if they take into account mothers feelings about length of hospital stay, as mothers who report ‘too short’ stays may feel unprepared to take care of their infant, even if they have lengthy objectively measured stays.

Lastly, having an infant who weighed 2500 g or more at birth was associated with difficulty accessing a health care provider for that infant. This may be because a shortage of pediatricians accepting infants in many areas of Canada has resulted in only infants who are in need of extensive follow-up care, such as those infants who are premature or small for gestational age, to be referred to pediatricians. Therefore, families with full term normal weight babies are likely to be responsible for finding their own primary health care provider, and thus more apt to experience difficulty.

While this study is based on data collected ten years ago, the characteristics we have identified as being related to difficulty accessing health care for an infant are likely to still apply. While the last ten years have seen provinces such as Ontario and Quebec undergo changes in the structure and payment models of many primary health care teams, there is little evidence that these changes have resulted in improved access. For example, a 2013 study based in Ontario compared frequency of accessing a primary care physician by low income patients in three payment models, the traditional salaried and Fee For Service (FFS) models (physician gets paid a certain amount for providing a certain service to a patient), and the newer capitation model (physician gets paid based on the number of patients in his or her practice adjusted for age and sex). The results suggest that after adjustment for factors such as age, rurality etc., low income patients in the capitation model saw their physicians less frequently and for smaller durations of time than patients in the FFS and salaried models [32]. This suggests that either patients in the capitation model have more difficulty accessing appointments or that physicians in the capitation models are less likely to accept patients that require more care. Both of these scenarios suggest decreased health care access for disadvantaged patients. In addition, there is little evidence that recent changes in structures of some primary health care teams (incorporating allied health professionals and more primary care providers in one team) increase accessibility. For example, prior to 2003 most family physicians in Quebec practiced in solo or group practices; however, beginning in 2003 the government began supporting the creation of Family Medicine Groups (FMG) that consist of 6 to 10 family physicians, and allied health professionals. To study equality of accessibility under this new model, a study based in two urban regions of Quebec compared affiliation with a family physician in the old (single or group practice) and new FMG model between 2005 and 2010. The results indicate that over this time period low income patients were increasingly more likely to be accepted into the old model and were increasingly less likely to be accepted as patients in the new FMG model [33]. Overall, these results are indicative of the fact that while the last 10 years have seen many Canadian provinces undergo changes in the structure and funding of primary health care teams, there is no evidence that health care access has improved or become more equitable.

### Strengths and limitations

This study had several strengths. First, the data’s representative nationwide scope is likely to allow the results to be generalizable widely across Canada, and gave us the ability to compare the prevalence rates of reporting difficulty across provinces. Second, the relatively large sample size allowed for ample statistical power. Third, confounding bias was minimized due to the wide variety of potential predictors that were controlled for in the analysis, including those that have never been examined in association with Canadian children’s or infants’ poor health care access. However, this study also has several limitations. First, mothers’ self-report that their infants had a non-routine problem or illness that needed to be seen by a health care provider, as well as the self-report of both the dependent and independent variables, may have resulted in misclassification bias. Second, due to the cross-sectional nature of the data, causal relationships cannot be inferred. Third, by excluding mothers living on First Nations Reserves and mothers who gave birth outside of Canada, the MES may have excluded severely socially and economically disadvantaged mothers, thereby reducing the generalizability of this analysis to these extremely disadvantaged groups.

## Conclusion

Our study suggests that ease of accessing health care for an infant may not be equitable under Canada’s universal health system. These disparities may lead to increased health inequality, contribute to the adverse health and development outcomes for infants of mothers with depressive symptoms, and lead to poor social integration and increased levels of stress for immigrant mothers. Given the adverse outcomes associated with inadequate health care access among infants, emphasis needs to be placed on reducing these disparities. Several simple interventions, such as providing immigrant mothers with information on how to navigate the Canadian health care system, providing referrals to health care providers for infants whose mothers report low levels of social support or depressive symptoms, and considering mothers feelings about length of postpartum hospital stay in after-birth follow up appointments may result in meaningful improvements in ease of health care access.
